# Long noncoding RNA LINC00707 sponges miR-370-3p to promote osteogenesis of human bone marrow-derived mesenchymal stem cells through upregulating WNT2B

**DOI:** 10.1186/s13287-019-1161-9

**Published:** 2019-02-22

**Authors:** Bo Jia, Zhiping Wang, Xiang Sun, Jun Chen, Jianjiang Zhao, Xiaoling Qiu

**Affiliations:** 1 Department of Oral Surgery, Stomatological Hospital, Southern Medical University, Guangzhou, 510280 China; 2 Department of General consulting and Emergency, Stomatological Hospital, Southern Medical University, 366 South Jiang Nan Road, Haizhu, Guangzhou, 510280 Guangdong People’s Republic of China

**Keywords:** Human mesenchymal stem cells, LINC00707, miR-370-3p, WNT family member 2B, Osteogenic differentiation

## Abstract

**Background:**

Human bone marrow-derived mesenchymal stem cells (HBMSCs) are characterized by multiple differentiation potential and potent self-renewal ability, yet much remains to be elucidated on what determines these properties. Long-chain noncoding RNAs (lncRNAs) have been suggested to be involved in multiple biological processes under physiological and pathological conditions, including osteogenic differentiation.

**Methods:**

Alkaline phosphatase (ALP) activity assay, ALP staining, and Alizarin Red Staining were used for osteogenic potential detection. Western blot and qRT-PCR were used to examine the expression of LINC00707 and miR-370-3p. RNA-binding protein immunoprecipitation was used to detect the interaction between LINC00707 and RNA-induced silencing complex. Luciferase reporter assay was used to confirm the binding sites of miR-370-3p to LINC00707 and WNT2B.

**Results:**

We demonstrated that LINC00707 expression was gradually increased in HBMSCs during consecutive osteogenic induction, and it could further positively regulate the osteogenic differentiation both in vitro and in vivo, whereas LINC00707 inhibition led to suppressed osteogenic differentiation. Thereafter, we inferred a predicted interaction between LINC00707 and miR-370-3p and then confirmed the direct binding sites of miR-370-3p on LINC00707. While miR-370-3p upregulation led to decreased osteogenic differentiation, LINC00707 overexpression could reverse this suppression, indicating that LINC00707 acts as a competing endogenous RNA (ceRNA) for miR-370-3p. Moreover, LINC00707 could act as a ceRNA to upregulate WNT2B via miR-370-3p inhibition.

**Conclusions:**

In conclusion, our study provides a novel lncRNA-miRNA regulatory network and a promising target to modulate the osteogenic differentiation of HBMSCs.

**Electronic supplementary material:**

The online version of this article (10.1186/s13287-019-1161-9) contains supplementary material, which is available to authorized users.

## Background

Periodontitis is an infectious and inflammatory oral disease that causes destruction of periodontal tissue and is a major cause of tooth loss [1]. Dentists can successfully control periodontal inflammation through conventional therapies but often cannot achieve recovery of damaged periodontal tissue. Until now, various stem cells have been investigated for periodontal regeneration, including mesenchymal stem cells (MSCs), embryonic stem cells, and induced pluripotent stem cells. Among these, MSCs are being increasingly accepted for periodontal regeneration because they are easy to acquire and culture [2]. As the most widely used and most studied cells [3, 4], MSCs can differentiate into bone marrow mesenchyme, teeth, alveolar bone, cementum, peripheral nerves, and blood vessels [5–8].

In recent years, with the discovery and study of noncoding RNAs (ncRNAs), the complex relationship between ncRNAs and disease has attracted great attention [9]. Long-chain noncoding RNA (lncRNA) is defined as an ncRNA longer than 200 nucleotides. Many studies have demonstrated that dysregulated expression of lncRNA is closely related to the diversity of multiple genes [10]. Recent studies have shown that the expression of certain lncRNAs may be involved in cell proliferation, differentiation, and osteogenesis [11]. MicroRNAs (miRNAs) are typically shorter than 22 nucleotides and can bind to a partially complementary sequence in the 3′ UTR of the target mRNA, causing translational inhibition [12, 13] and/or mRNA destabilization by Argonaute (Ago) proteins. Recent studies have found that, as pseudogenes, lncRNAs can act as miRNA “sponges” by sharing common microRNA recognition elements (MREs), thereby inhibiting normal miRNA activity [14].

WNT2B is an important protein in the WNT/β-catenin signaling pathway [15], which is itself a classical signaling pathway in osteogenic differentiation of bone marrow stromal cells (BMSCs) [16] and plays an important regulatory role in the differentiation of human BMSCs [17]. The WNT/β-catenin signaling pathway regulates the expression of osteoblast-associated genes runt-related transcription factor 2 (RUNX2) and Osterix (OSX) at the transcriptional level [18]. Osterix acts as a downstream target gene of RUNX2, and both of them play a major regulatory role in osteoblast differentiation [19]. WNT2B can regulate the expression of RUNX2 and promote Osterix expression at the gene and protein levels. After siRNA interference, it significantly inhibits the expression of alkaline phosphatase (ALP), indicating that WNT2B can regulate the expression of key genes of osteogenesis, such as RUNX2 and Osterix, and thus regulate the process of bone formation. Studies have shown that WNT2B may promote osteogenesis of human bone marrow-derived mesenchymal stem cells (HBMSCs) [20].

In this study, LINC00707 was found to be involved in the process of osteogenic differentiation of BMSCs. Bioinformatics prediction methods indicated that miR-370-3p can be bound by LINC00707, and thus upregulate the expression of its target gene WNT2B. To our knowledge, this is the first report to describe the relationship between the LINC00707/miR-370-3p axis and osteogenic differentiation of HBMSCs.

## Materials and methods

### Cell cultures

Human bone marrow-derived mesenchymal stem cells (HBMSCs) were purchased from SALILA (Guangzhou, China), the HBMSCs were isolated and purified from fresh human bone marrow aspirates donated from the iliac crest of healthy adults less than 40 years of age. The cells were cultured in Dulbecco’s modified Eagle’s medium (Gibco, Carlsbad, CA) supplemented with 10% fetal bovine serum at 37 °C in a humidified atmosphere of 5% CO_2_. For osteogenic induction, HBMSCs were cultured with osteogenic differentiation medium (Cyagen, Guangzhou, China) with 100 mmol/L dexamethasone, 0.05 mmol/L ascorbic acid, and 10 mmol/L β-glycerophosphate. The medium was changed every 2 days.

### Flow cytometry

P6 HBMSCs were digested to single-cell suspensions, washed three times in PBS, adjusted to the 10^6^ cells per sample, and stained for CD29, CD44, and CD90. The antibodies specific for human molecules were used as follows: FITC-CD29 (Beckman Coulter, USA), PE-CD44 (Beckman Coulter), APC-CD90 (Beckman Coulter, USA). Flow cytometry was performed on the Moflo XDP (Beckman Coulter, Brea, CA, USA). The corresponding isotype control monoclonal antibodies were purchased from Beckman Coulter.

### lncRNA sequencing analysis

Three pairs of osteogenic differentiation induced HBMSCs, and the paired non-induced HBMSCs were used to perform RNA sequencing at the BGI (Shenzhen, China). Ribo-Zero™ rRNA Removal Kits (Epicentre, Madison, WI, USA) were used for rRNA depletion. Paired-end, strand-specific reads of 91 nt were generated using an Illumina HiSeqTM2000, and the mapped reads were assembled by Cufflinks 2.0.2. Differential expression lncRNAs with statistical significance was performed with the EdgeR package on R, and cutoffs were established at a fold change > 2 and a *P* value < 0.05.

### Quantitative real-time PCR

Total RNA was extracted from cells using TRIzol reagent (Invitrogen, Carlsbad, CA, USA), and reverse-transcription reactions were performed using random primers and an M-MLV Reverse Transcriptase kit (Invitrogen). Real-time PCR was performed using a standard SYBR Green PCR kit (Toyobo, Osaka, Japan) protocol on an Applied Biosystems 7300 Real-Time PCR system (Applied Biosystems, Foster City, CA, USA) according to the manufacturer’s instructions. GAPDH was used as reference for lncRNAs and mRNAs; U6 was used as reference for miRNAs. The 2^−ΔΔCt^ method was used to determine the relative quantitation of gene expression levels. Each sample was analyzed in triplicate. Primer sequences are listed in Additional file 1: Table S1.

### Western blotting

Total protein was extracted from the cells by lysis with radioimmunoprecipitation assay buffer (DGCS Biotechnology, China). The protein content of the lysate was then determined using the BCA Protein Assay Kit (Beyotime, China) according to the manufacturer’s protocol. Cell lysates were resolved on 10% sodium dodecyl sulfate-polyacrylamide gel electrophoresis and transferred onto a polyvinylidene fluoride membrane (Millipore, Billerica, MA). The membrane was blocked with TBS-T (0.1% Tween-20 in TBS) containing 5% BSA, and incubated with primary antibody overnight at 4 °C. After three washes with TBS-T, the membrane was incubated with HRP-conjugated secondary antibody for 1 h and then washed with TBS-T. Immune complexes were detected by chemiluminescence (SuperSignal West Femto; Pierce Biotechnology, Waltham, MA) using a MyECL Imager (Thermo Scientific, Waltham, MA). Mean densitometry data from independent experiments were normalized to the control. Primary antibodies are listed as follows: Runx2 (1:1000, Cell Signaling Technology, Danvers, MA), GAPDH (1:1000, Cell Signaling Technology), WNT2B (1:800, Abcam, Cambridge, UK), osteocalcin (OCN) (1:1000, Abcam, Cambridge, UK), ALP (11,000, Abcam, Cambridge, UK). All experiments were performed in triplicate.

### ALP activity assay

Transfected HBMSCs were cultured with an osteogenic induction medium for 7 days in 24-well plates at a density of around 1 × 10^5^ cells per well, and then ALP activity was measured using the ALP activity kit according to the manufacturer’s protocol (Nanjing Jiancheng Bioengineering Institute, Jiangsu Sheng, China).

### Mineralization assay

Transfected HBMSCs were cultured in a 24-well plate with an osteogenic induction medium for 7 days at a density of about 1 × 10^5^ cells per well, and mineralization of HBMSCs was observed using Alizarin Red S (ARS) and ALP staining with manufacturing agreement (ARS, Solarbio, Beijing, China; ALP Staining Kit, Nanjing).

### Hematoxylin and Eosin (HE) and Masson’s trichrome staining

NOD/SCID mice (about 4 weeks old) purchased from the Provincial Animal Center (Guangdong, China) were randomly divided into indicated groups of six mice per group. For transplantation, approximately 5 × 10^6^ cells were loaded onto 10 mg hydroxyapatite/tricalcium phosphate (HA-TCP; Sigma, St Louis, MO) and subcutaneously implanted into the dorsal region of NOD/SCID mice. For all surgical procedures, general anesthesia was administered by intramuscular injection of sodium pentobarbital (0.1 mL/100 g body weight). All animal procedures were approved by the Animal Care Committee of Southern Medical University. After 4 weeks, the mice were sacrificed by cervical dislocation under general anesthesia. Xenografts were removed, fixed with 4% paraformaldehyde, and decalcified in 10% EDTA (pH 6.0) for 7 days. For histological analysis, xenografts were embedded in paraffin, sectioned, and stained with HE or Masson’s Trichrome stain (Sigma) according to the manufacturers’ protocols.

### Luciferase reporter assay

Putative miR-370-3p target binding sequence in wild-type and mutant LINC00707 and WNT2B were synthesized and cloned downstream of the luciferase gene in the pmirGLO luciferase vector (GeneChem, Shanghai, China). Plasmids used as standardized controls, miRNA mimics (miR-NC and miR-370-3p), and dual-luciferase plasmid (GeneChem) were commonly transfected into cells using Lipofectamine 2000 (Invitrogen). Firefly and Renilla luciferase activities were then continuously measured using dual luciferase assay 48 h after transfection. All experiments were performed in triplicate.

### Lentiviruses and cell transfection

The shRNA against LINC00707 and scrambled shRNA were synthesized and cloned into the pGLVU6/GFP vector, named sh-LINC00707 and sh-NC. The LINC00707 sequence was amplified and inserted into the vector for LINC00707 overexpression, a blank plasmid vector as an NC. This work was provided by GeneChem (Shanghai GeneChem, Shanghai, China). For lentivirus packaging, we transferred 293T cells with the plasmid vector along with the packaging plasmids Helper 1.0 and Helper 2.0 (Shanghai GeneChem). HBMSCs were cultured in six-well plates and transfected with lentivirus in the presence of polybrene at a multiplicity of transfection of 50 when the cells had reached 30–40% confluence.

### Oligonucleotide, plasmids, and cell transfection

siRNAs, miRNA mimics, and miRNA inhibitors were purchased from RiboBio (Guangzhou, China); plasmids were purchased from GeneChem. These reagents were transfected into HBMSCs at final concentrations of 50 nM for miRNA and 25 nM for siRNA in a six-well plate using Lipofectamine 3000 transfection reagent (Invitrogen) following the manufacturer’s protocol. After 48 h of transfection, the cells were collected and used for further analysis.

### RNA immunoprecipitation (RIP)

RIP assay was performed using an RNA Binding Protein Immunoprecipitation Kit (Millipore) with anti-Ago2 antibody (1:1000, Abcam) or normal mouse IgG as a negative control according to the manufacturer’s protocol. qPCR was then performed as described above. All experiments were performed in triplicate.

### Statistical analysis

Data are expressed as the mean ± standard deviation (SD) from at least three separate experiments. Differences between groups were analyzed using Student’s *t* test (when two groups were compared) or analysis of variance followed by Bonferroni’s test (when > 2 groups were compared). Two-tailed Pearson’s correlation analysis was used to evaluate the association of the two variables. A value of *P* < 0.05 was deemed statistically significant.

## Results

### High-throughput sequencing analysis of lncRNA after HBMSC-induced osteogenic differentiation for 15 days

**Fig. 1:**
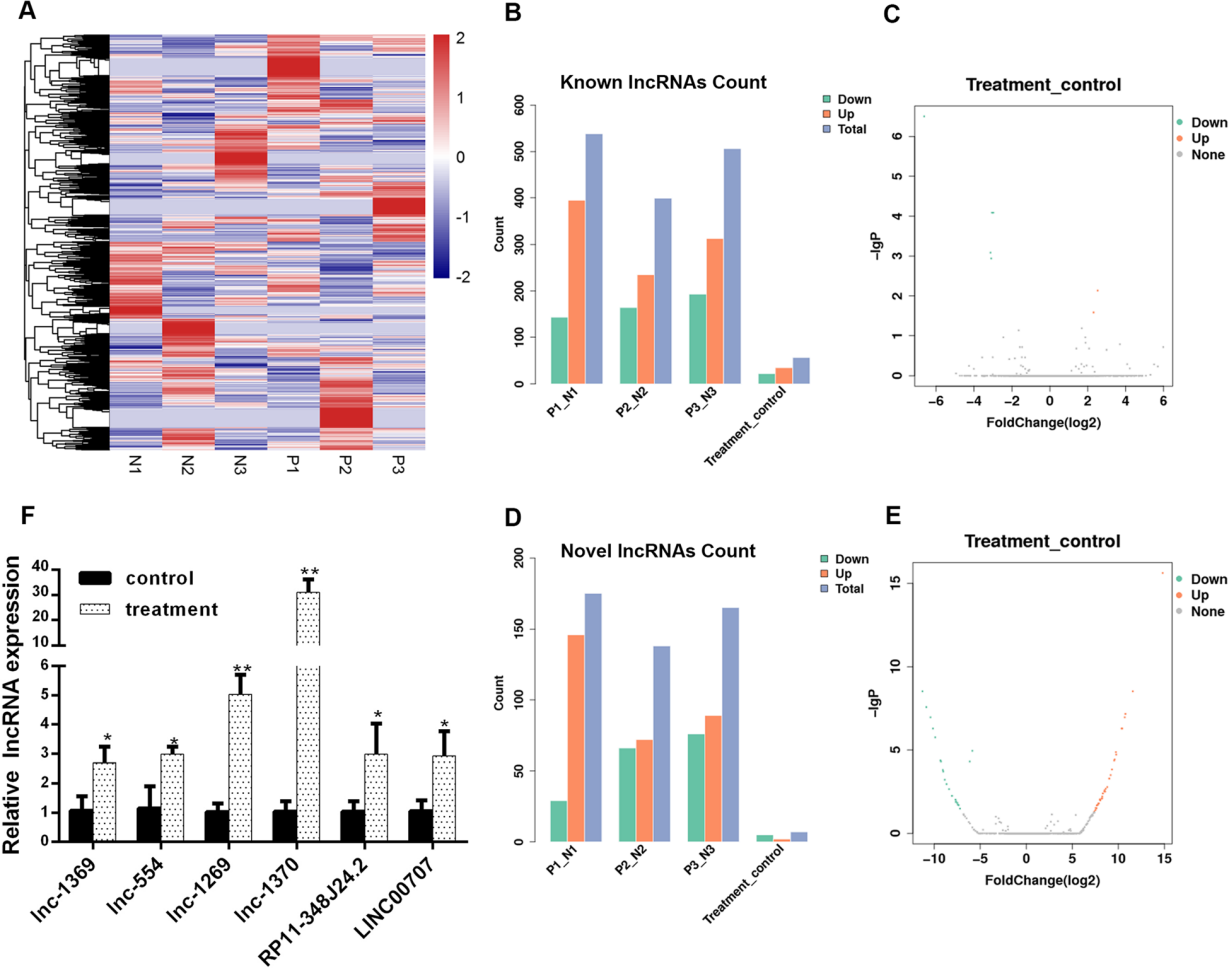
High-throughput sequencing analysis of lncRNA in HBMSCs treated with osteogenic-induced medium for 15 days. **a** Heatmap of differentially expressed lncRNAs after osteogenic differentiation induction. **b** Counts of known differentially expressed lncRNAs in each group. **c** Volcano plot of known differentially expressed C. **d** Counts of unknown differentially expressed lncRNAs in each group. **e** Volcano plot of unknown differentially expressed lncRNAs. **f** qRT-PCR validation of known and unknown lncRNAs with the largest fold change. Data represent mean ± S.D. *P<0.05, **P<0.01

Flow cytometry revealed that P6 HBMSCs also expressed CD29 (98.9%), CD44 (98.1%), and CD90 (97.6%), which indicated the HBMSCs were not contaminated with cells of heterogeneity of the populations (Additional file 1: Figure S1). To induce osteogenic differentiation, P3 HBMSCs were treated with osteogenic differentiation medium. High-throughput sequencing was applied to detect the lncRNA expression levels before and after osteogenic differentiation. A heatmap describing the changes in lncRNAs is shown in Fig. 1a. Upregulated and downregulated lncRNAs in known lncRNAs and novel lncRNAs were counted (Fig. 1b, d) and volcano plots of known lncRNAs and novel lncRNAs were shown (Fig. 1c, e). We selected six obviously changed lncRNAs (Fig. 1f) for verification in HBMSCs.

### LINC00707 expression is correlated with osteogenic differentiation of HBMSCs

**Fig. 2:**
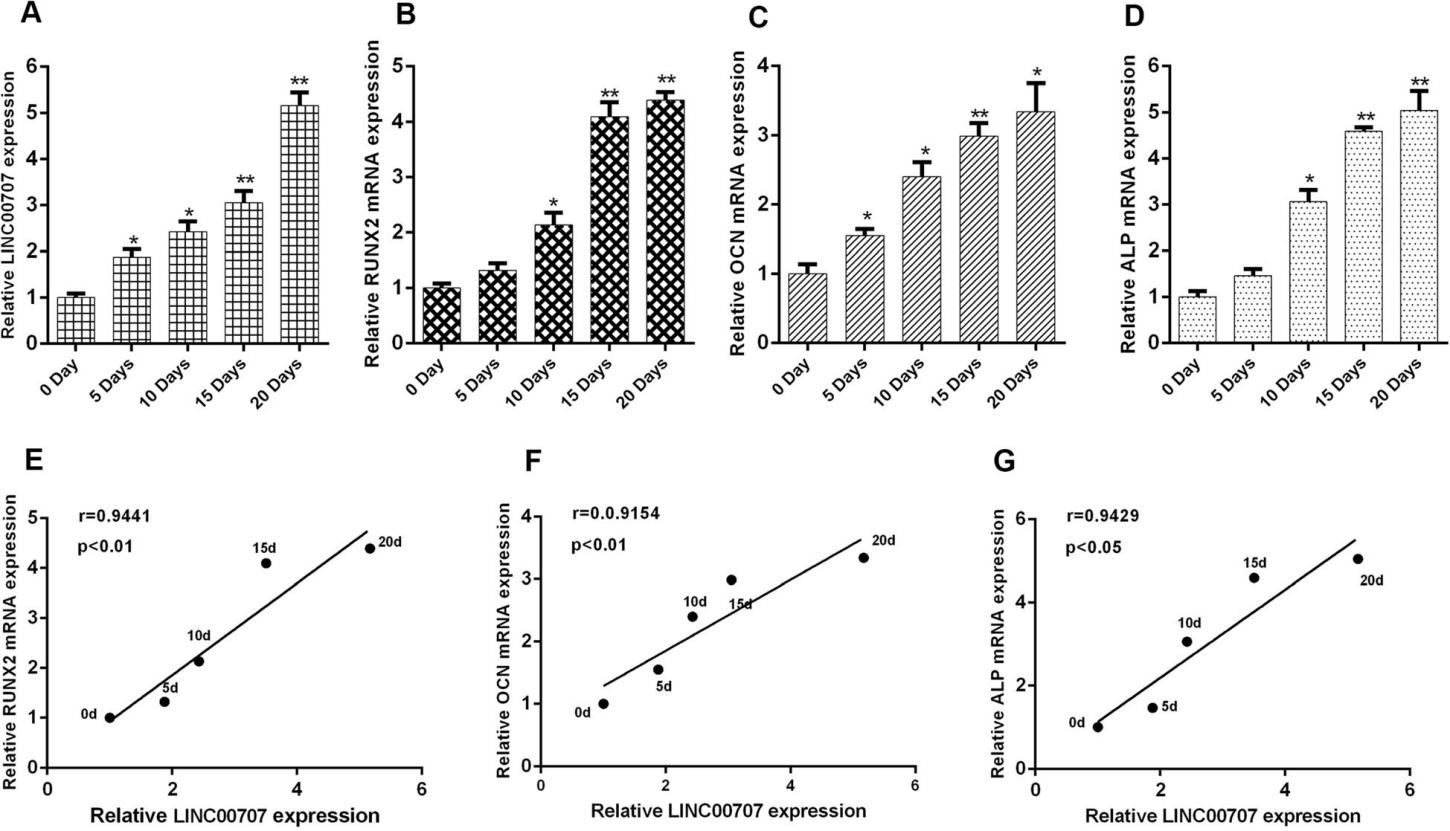
Expression of LINC00707 is correlated with osteogenic differentiation of HBMSCs. **a**–**d** Expression of lnc LINC00707, RUNX2, OCN, and ALP at 0, 5, 10, 15, and 20 days were assayed by qRT-PCR after osteogenic induction of HBMSCs. **e**–**g** Correlation between LINC00707 expression and osteogenesis-related genes expression during osteogenic induction. Data represent the mean ± S.D. *P<0.05, **P<0.01

The expression levels of LINC00707 and three representative osteogenic genes, namely RUNX2, OSX, and ALP, were determined in HBMSCs at 0, 5, 10, 15, and 20 days after osteogenic induction. They were all found to be gradually increasing during induction (Fig. 2a–d), and an obviously positive correlation between the expression levels of LINC00707 and the three representative osteogenic genes were determined (Fig. 2e–g). This led us to speculate on the potential role of LINC00707 in osteogenic induction.

### LINC00707 stimulates osteogenic differentiation of HBMSCs in vitro and in vivo

**Fig. 3:**
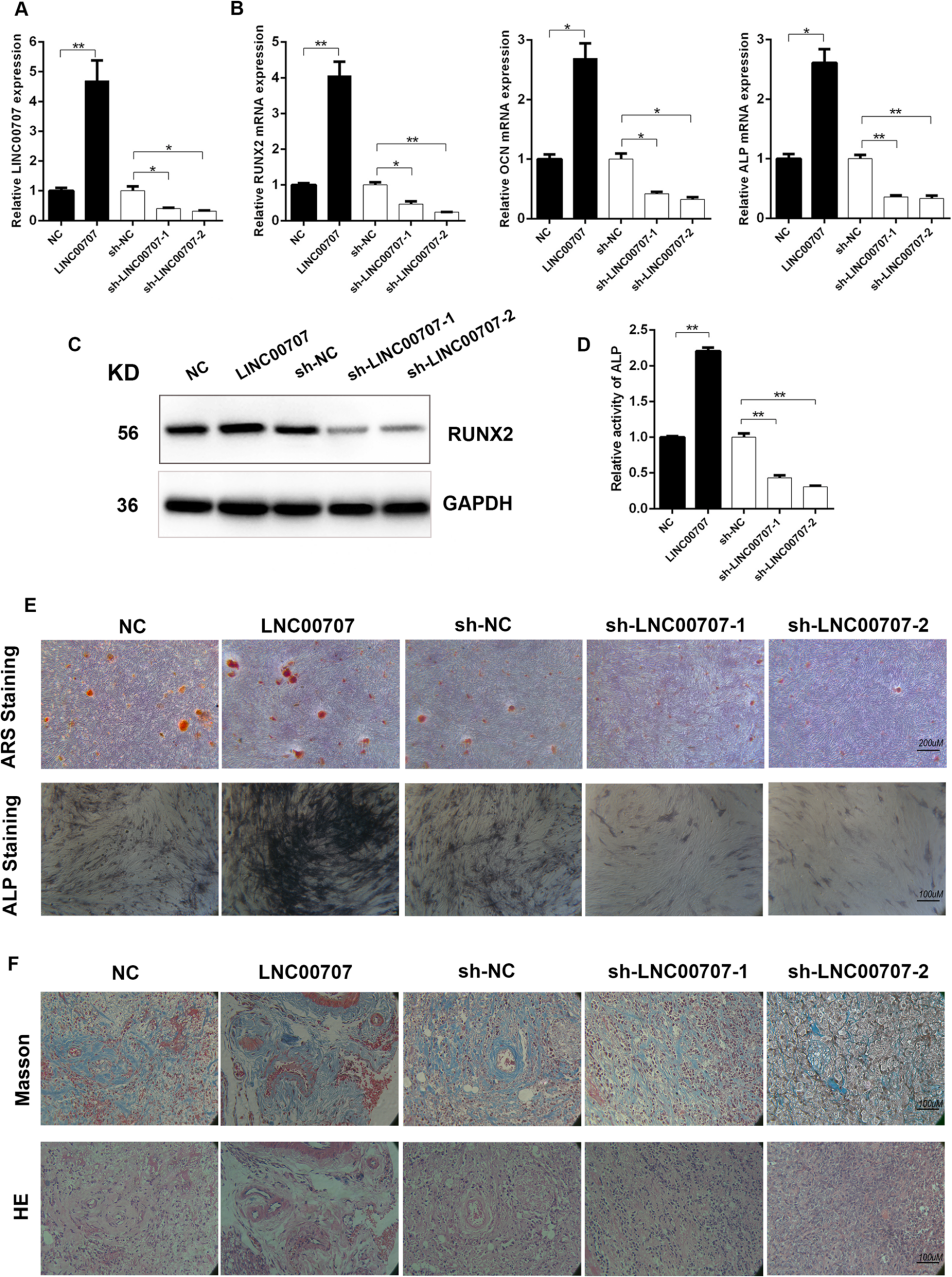
LINC00707 stimulates osteogenic differentiation of HBMSCs in vitro and in vivo. **a** Efficiency of interference and overexpression of LINC00707 in HBMSCs by qRT-PCR. **b** mRNA levels of RUNX2, OCN, and ALP in transfected HBMSCs after 7 days of osteogenic induction by qRT-PCR. **c** Western blotting analysis of protein level of RUNX2 in transfected HBMSCs after 7 days osteogenic induction. **d** Relative activity of ALP after interference or overexpression of LINC00707 in transfected HBMSCs after 7 days osteogenic induction. **e** ARS staining and ALP staining after interference or overexpression of LINC00707 in transfected HBMSCs after 7 days osteogenic induction. **f** Transfected HBMSCs after 7 days osteogenic differentiation loaded on HA-TCP were transplanted into the dorsal region of NOD/SCID mice. Xenografts were removed and stained with HE and Masson’s Trichrome stain, respectively. Data represent the mean ± S.D. *P<0.05, **P<0.01

To determine the effect of LINC00707 on osteogenic differentiation of HBMSCs, we constructed LINC00707-overexpressing and LINC00707-knockdown HBMSCs using lentiviruses. LINC00707 expression levels were confirmed using qRT-PCR (Fig. 3a). From previous research, LINC00707 has positive correlations with ALP, RUNX2, and OCN, and so we assessed the mRNA expression levels of ALP, RUNX2, and OCN after 7 days of osteogenic induction. We found that LINC00707 overexpression markedly upregulated the mRNA levels of ALP, RUNX2, and OCN. However, LINC00707 knockdown significantly downregulated the expression of ALP, RUNX2, and OCN mRNA (Fig. 3b). LINC00707 was further found to also regulate RUNX2 at the protein level (Fig. 3c). To further investigate the effects of LINC00707 on the osteogenic process, ALP activity assay, ARS staining, and ALP staining were performed and revealed that LINC00707 overexpression increased ALP activity and mineralized bone matrix formation in HBMSCs, whereas these were significantly decreased upon LINC00707 knockdown (Fig. 3d, e and Additional file 1: Figure S2). Next, we further evaluated the effect of LINC00707 on HBMSC osteogenesis in vivo. Transfected HBMSCs loaded with HA-TCP were implanted subcutaneously in the dorsal region of NOD/SCID mice. After 4 weeks, the implants were removed and the newly formed bone tissue was then stained with HE and Masson’s Trichrome. LINC00707 overexpression increased osteoid formation whereas knockdown of LINC00707 resulted in decreased osteoid formation compared with the negative control (Fig. 3f). In summary, the results showed that LINC00707 could promote osteogenic differentiation of HBMSCs both in vitro and in vivo.

### Analysis of the combination of LINC00707 and miR-370-3p

**Fig. 4:**
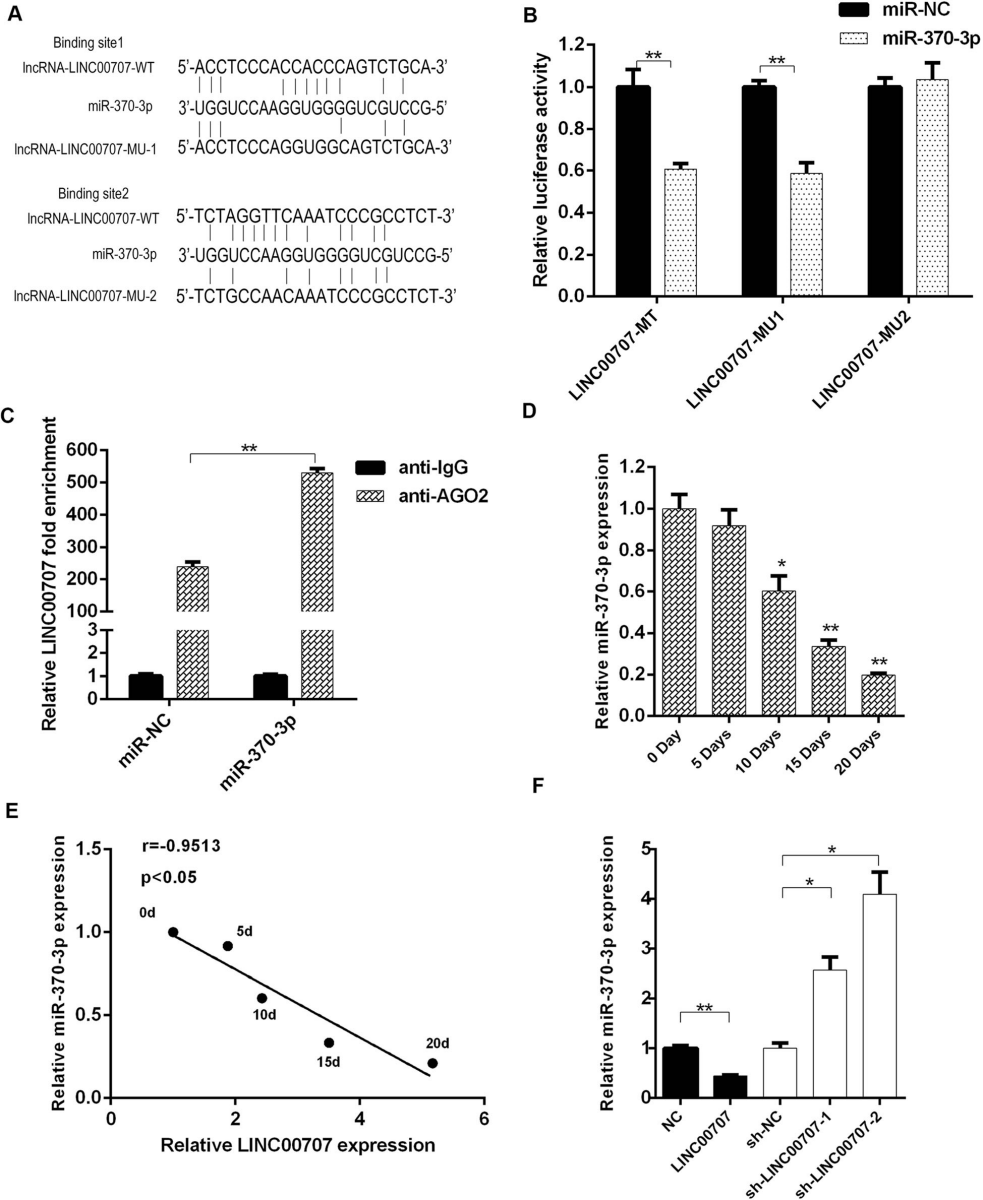
Analysis of the combination of LINC00707 and miR-370-3p. **a** Schematic diagram of miR-370-3p putative binding sites in wild type and mutant LINC00707. **b** Dual-luciferase reporter assay to detect the binding sites between LINC00707 and miR-370-3p. **c** Ago2-RIP to verify the binding of lncC00707 to miR-370-3p. **d** miR-370-3p level determined in HBMSCs at 0, 5, 10, 15, and 20 days after osteogenic induction by qRT-PCR. **e** Correlation analysis was performed between miR-370-3p and LINC00707 levels at indicated times after osteogenic induction. **f** miR-370-3p level following interference and overexpression of LINC00707 was determined by qRT-PCR. Data represent the mean ± S.D. *P<0.05, **P<0.01

To determine whether LINC00707 acts as an miRNA sponge that competes with mRNA for binding to miRNAs, we used LncRNABase and NONCODE to find two putative binding sites of miR-370-3p to LINC00707 (Fig. 4a). Based on this prediction, dual-luciferase reporter constructs carrying a LINC00707 reporter were generated. The results showed that the LINC00707-wt reporter and LINC00707-mut1-reporter were strongly inhibited by miR-370-3p, while the LINC00707-mut2 reporter was not affected by miR-370-3p (Fig. 4b), indicating that miR-370-3p binds directly to LINC00707 at the predicted binding site 1. RIP assay was performed to confirm that LINC00707 is associated with the RNA-induced silencing complex, indicating the potential relationship between LINC00707 and miR-370-3p (Fig. 4c). In addition, miR-370-3p in HBMSCs gradually decreased during osteogenic induction (Fig. 4d). Considering a negative correlation between miR-370-3p expression and LINC00707 expression during osteogenic induction, we further evaluated miR-370-3p level in HBMSCs and found a decrease following LINC00707 overexpression (Fig. 4e). Therefore, LINC00707 silencing resulted in miR-370-3p upregulation in HBMSCs.

### LINC00707 negatively regulates miR-370-3p during osteogenic induction

**Fig. 5:**
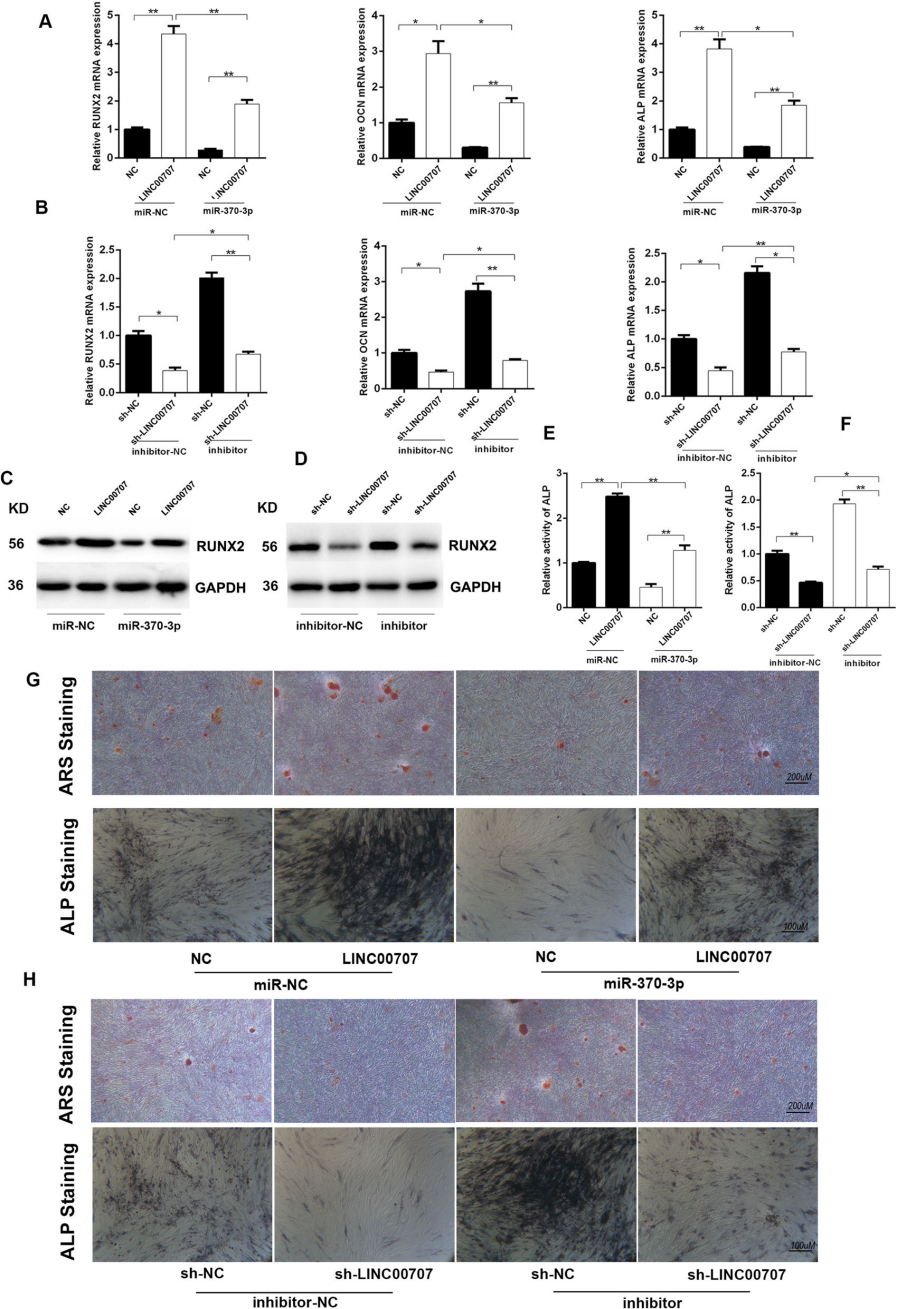
LINC00707 negatively regulate miR-370-3p during osteogenic induction **a** mRNA levels of RUNX2, ALP, and OCN detected by qRT-PCR in HBMSCs co-transfected with LINC00707/NC and miR-370-3p mimics/mimics NC after osteogenic induction for 7 days. **b** mRNA levels of RUNX2, ALP, and OCN were detected by RT-PCR in HBMSCs co-transfected with sh-LINC00707/sh-NC and miR-370-3p inhibitor/inhibitor NC after osteogenic induction for 7 days. **c** Protein level of RUNX2 in HBMSCs co-transfected with LINC00707/NC and miR-370-3p mimics/mimics NC after osteogenic induction for 7 days. **d** Protein level of RUNX2 in HBMSCs co-transfected with sh-LINC00707/sh-NC and miR-370-3p inhibitor/inhibitor NC after osteogenic induction for 7 days. **e**, **g** Relative activity of ALP, ARS staining, and ALP staining in HBMSCs co-transfected with LINC00707/NC and miR-370-3p mimics/mimics NC after osteogenic induction for 7 days. **f**, **h** Relative activity of ALP, ARS staining, and ALP staining in HBMSCs co-transfected with sh-LINC00707/sh-NC and miR-370-3p inhibitor/inhibitor NC after osteogenic induction for 7 days. Data represent the mean ± S.D. *P<0.05, **P<0.01

It has previously been reported that miR-370-3p was associated with osteogenic differentiation. Since LINC00707 could act as a sponge for miR-370-3p, we then investigated how this interaction affects osteogenic differentiation. Combining the preceding findings, miR-370-3p mimics decrease RUNX2, ALP, and OCN mRNA levels in LINC00707-overexpressing HBMSCs whereas miR-370-3p inhibitor increased these levels in LINC00707-knockdown HBMSCs (Fig. 5a, b). Regulation of the LINC00707/miR-370-3p axis in RUNX2 was further confirmed at the protein level (Fig. 5c, d). The LINC00707/miR-370-3p axis can also regulate ALP activity (Fig. 5e, f). In addition, ARS and ALP staining were used to evaluate the mineralization in co-transfected HBMSCs after osteogenic induction, and negative regulation between LINC00707 and mir-370-3p was observed (Fig. 5g, h and Additional file 1: Figure S3).

### WNT2B is a target of LINC00707/miR370-3p axis during osteogenesis

**Fig. 6:**
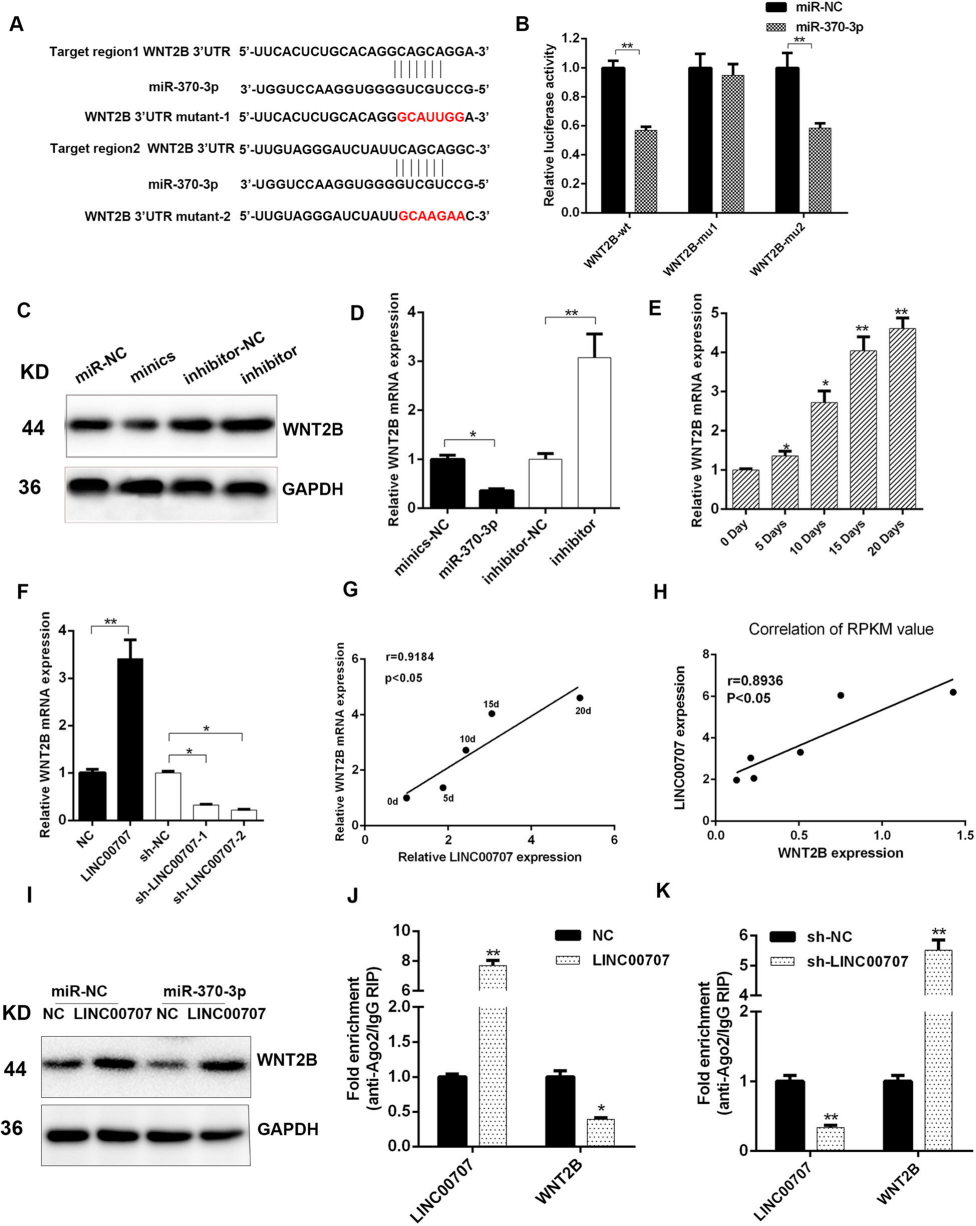
WNT2B is a LINC00707/miR370-3p axis target during osteogenesis. **a** Schematic diagram of miR-370-3p putative binding sites in wild type and mutant WNT2B 3′-UTR. **b** Binding sites of miR-370-3p to wild type or mutant WNT2B 3′UTR under transfection with miR-370-3p mimics were determined with dual-luciferase reporter assay. **c** WNT2B protein level determined in HBMSCs transfected with miR-370-3p mimics or inhibitor using Western blotting. **d** WNT2B mRNA level determined in HBMSCs transfected with miR-370-3p mimics or inhibitor using qRT-PCR. **e** WNT2B mRNA level was determined in HBMSCs at 0, 5, 10, 15, and 20 days after osteogenic induction by qRT-PCR. **f** WNT2B mRNA level was determined in LINC00707-overexpressing or LINC00707-silenced HBMSCs. **g** Correlation analysis was performed between miR-370-3p and LINC00707 levels at indicated times after osteogenic induction. **h** Correlation analysis was performed between miR-370-3p and LINC00707 RPKM values tested by high-throughput sequencing analysis after osteogenic induction. **i** WNT2B expression levels detected by Western blot assay in HBMSCs, co-transfected with LINC00707/NC and miR-370-3p mimics/mimics-NC after osteogenic induction for 7 days. **j**, **k** RIP assay of enrichment of Ago2 on LINC00707 and WNT2B transcripts relative to IgG in HBMSCs transfected with LINC00707-overexpressing or LINC00707-silenced lentivirus and their corresponding controls. Data represent the mean ± S.D. *P<0.05, **P<0.01

TargetScan and microT-CDS were used for analysis of the targets of miR-370-3p. Among the common predicted target genes, WNT2B was also an upregulated gene on high-throughput sequencing result in this study that was previously reported to participate in osteogenic differentiation. Following the above findings, dual-luciferase reporter assay was used to confirm the binding site of miR-370-3p in WNT2B (Fig. 6a, b). Protein and mRNA levels of WNT2B were found to be decreased in HBMSCs transfected with miR-370-3p mimics, but were increased by the miR-370-3p inhibitor (Fig. 6c, d). Consistently, WNT2B mRNA levels gradually increased in HBMSCs during continuous osteogenic induction (Fig. 6e) and negatively correlated with the miR-370-3p level (Fig. 6g, h). Protein and mRNA levels of WNT2B were increased in HBMSCs overexpressing LINC00707, but were decreased by LINC00707 silencing (Fig. 6f, i). Furthermore, overexpression of LINC00707 in HBMSCs led to increased enrichment of Ago2 in LINC00707 but substantially decreased enrichment in WNT2B transcripts. In parallel, LINC00707 knockdown elicited a significant increase in the recruitment of Ago2 to WNT2B transcripts compared with control cells. These results suggested that LINC00707 could compete with WNT2B transcripts for the Ago2-based miRNA-induced repression complex (Fig. 6j, k). In summary, the LINC00707/miR370-3p axis regulated WNT2B in HBMSCs during osteogenesis.

### Effect of WNT2B on osteogenesis

**Fig. 7:**
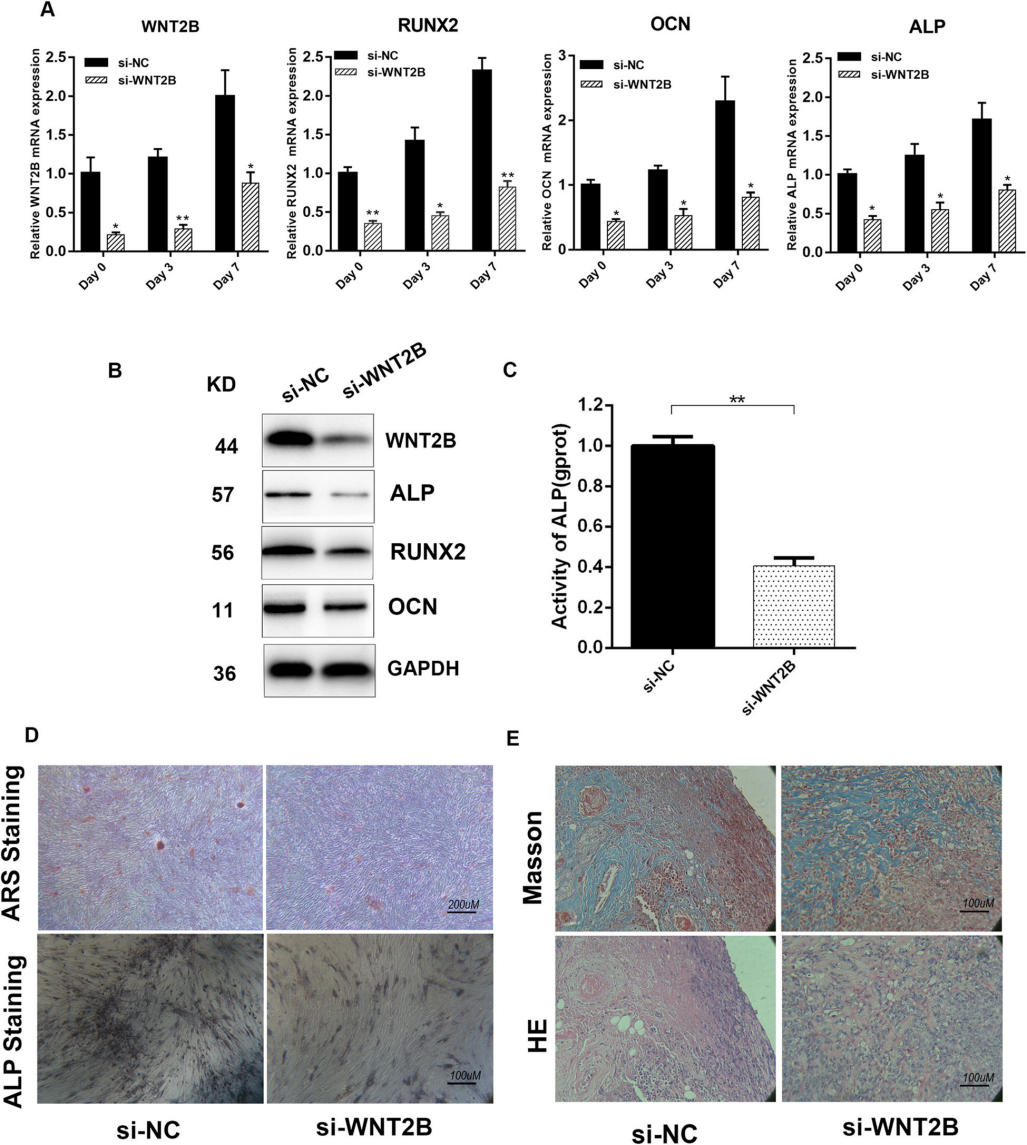
Effect of WNT2B on osteogenic induction. **a** mRNA levels of WNT2B, RUNX2, OCN, and ALP in HBMSCs transfected with si-WNT2B at 3 and 7 days after osteogenic induction. **b** Protein levels of WNT2B, RUNX2, OCN, and ALP in HBMSCs transfected with si-WNT2B after oncogenic induction for 7 days. **c** Activity of ALP in HBMSCs transfected with si-WNT2B after osteogenic induction for 7 days. **d** ARS staining and ALP staining in HBMSCs transfected with si-WNT2B after osteogenic induction for 7 days. **e** HE and Masson Trichrome staining were performed in WNT2B silenced HBMSCs in vivo. Data represent the mean ± S.D. *P<0.05, **P<0.01

It has been shown that WNT2B expression gradually increased during the process of osteogenesis. The effect of WNT2B silencing was therefore verified using qRT-PCR. Related representative osteogenesis genes, including RUNX2, OCN, and ALP were all downregulated in HBMSCs at 3 and 7 days after WNT2B silencing (Fig. 7a). The effect of WNT2B on the expression of RUNX2, OCN, and ALP was further confirmed at the protein level (Fig. 7b). To further investigate the effects of WNT2B on the osteogenic process, ALP activity assay, ARS staining, and ALP staining were performed and revealed that WNT2B knockdown decreased ALP activity and mineralized bone matrix formation in HBMSCs (Fig. 7c, d and Additional file 1: Figure S4). We then evaluated the effect of WNT2B on HBMSC osteogenesis in vivo further. Transfected HBMSCs loaded with HA-TCP were subcutaneously implanted in the dorsal region of NOD/SCID mice. After 4 weeks, the implants were removed and the newly formed bone tissue was then stained with HE and Masson’s Trichrome. WNT2B knockdown resulted in decreased osteoid formation compared with the negative control (Fig. 7e). In summary, these results indicated that WNT2B could promote osteogenic differentiation of HBMSCs both in vitro and in vivo.

## Discussion

Periodontitis is an infectious and inflammatory oral disease that causes destruction of periodontal tissue and is a major cause of tooth loss [20, 21]. Dentists have been able to successfully control periodontal inflammation using conventional therapies, but often cannot achieve recovery of damaged periodontal tissue. HBMSCs are easy to acquire and culture [22, 23], and thus are the most widely used and most studied MSCs. These cells can differentiate into bone marrow mesenchyme, alveolar bone, cementum, peripheral nerves, and blood vessels [24]. In the present study, we demonstrated increasing levels of LINC00707 during osteogenic differentiation and subsequent experiments revealed promotion of LINC00707 in osteogenic differentiation using gain-of-function and loss-of-function experiments. Histological assessment including HE and Masson staining was used to investigate bone regeneration, the newly formed bone tissue from HBMSCs differentiation was stained blue was significantly higher in implants containing LINC00707 overexpression cells than in negative controls. The above results indicate the potential role of LINC00707 in osteogenesis regulation in HBMSCs.

Studies have shown that LINC00707 is a non-coding oncogene that plays an important role in promoting tumor proliferation and migration. Ma et al. found that LINC00707 promotes proliferation and migration of lung adenocarcinoma cells by regulating Cdc42 [25]. It has also been reported that miR-370-3p can participate in the WNT signaling pathway [26]. Surprisingly, no studies have considered the effects of LINC00707 and miR-370-3p on osteogenic differentiation. Our study is the first to explore the possibility that LINC00707 can promote osteogenic differentiation of HBMSCs. Taking into account the sponge effect of lncRNAs, we further explored the potential relationship between LINC00707 and miR-370-3p. Our results showed that during the osteogenic induction process, increasing expression of LINC00707 with decreasing expression of miR-370-3p was verified. Luciferase reporter assay also showed that LINC00707 could sponge miR-370-3p and modulate its effects on osteogenic induction, indicating the possible involvement of a lncRNA-miRNA regulatory network during osteogenic differentiation.

WNT2B is an important member of the WNT family [27]. WNT family proteins play a major role in a variety of developmental processes, including regulation of cell growth and differentiation and the osteogenic differentiation process [28]. The WNT/β-catenin signaling pathway is a classical signaling pathway in the differentiation of HMSCs and plays an important regulatory role in their differentiation, especially in the process of osteoblast differentiation [28, 29]. Huang et al. showed that miR-370-3p has the ability to inhibit bladder cancer cell invasion [26]. Also, Li et al. reported that miR-370-3p can inhibit the process of epithelial–mesenchymal transition [30]. In our study, bioinformatics technology predicted that miR-370-3p could bind to the 3′ UTR of WNT2B, and negative regulation of miR-370-3p on WNT2B was demonstrated using the dual luciferase reporter gene for the first time. Furthermore, WNT2B was found to promote osteogenic differentiation of HBMSCs.

This study explored the effect of the LINC00707/miR-370-3p/WNT2B axis on osteogenic differentiation of HBMSCs. It is hoped that the LINC00707/miR-370-3p/WNT2B axis can be used to promote osteoblast differentiation of HBMSCs, and the HBMSCs can be applied in periodontal regeneration to restore damaged periodontal tissue.

## Conclusions

Considering a minority of the ncRNAs have been functionally determined, the regulation of biological functions by lncRNA-miRNA interaction networks may be far more important as we previously considered. In our study, we identified the regulation of osteogenic potential of BMSCs composed of LINC00707/miR-370-3p/WNT2B axis. Our study exhibited the complicated regulation within ncRNA and provided a promising target to regulate the osteogenic potential of HBMSCs for treatment of osteogenesis disorders.

## Additional file

Additional file 1: **Table S1.** Sequences of PCR primers used in this study. **Figure S1.** Flow cytometry analysis of HBMSCs. **Figure S2.** Quantitative analysis of ALP staining and ARS staining after interference or overexpression of LINC00707 in transfected HBMSCs after 7 days osteogenic induction. **Figure S3.** (A and B) Quantitative analysis of ARS staining and ALP staining in HBMSCs co-transfected with LINC00707/NC and miR-370-3p mimics/mimics NC after osteogenic induction for 7 days. (C and D) ARS staining and ALP staining in HBMSCs co-transfected with LINC00707/NC and miR-370-3p mimics/mimics NC after osteogenic induction for 7 days. **Figure S4.** ARS staining and ALP staining in HBMSCs transfected with si-WNT2B after oncogenic induction for 7 days. (DOCX 511 kb)

## Abbreviations

Ago: Argonaute
ALP: Alkaline phosphatase
BMSCs: Bone marrow stromal cells
HA-TCP: Hydroxyapatite/tricalcium phosphate
HBMSCs: Human bone marrow-derived mesenchymal stem cells
HE: Hematoxylin and Eosin
lncRNA: Long-chain noncoding RNA
miRNAs: MicroRNAs
MSCs: Mesenchymal stem cells
OCN: Osteocalcin
OSX: Osterix
RUNX2: Runt-related transcription factor 2
WNT2B: WNT family member 2B
