# Supplementary material for: Long noncoding RNA LINC00707 sponges miR-370-3p to promote osteogenesis of human bone marrow-derived mesenchymal stem cells through upregulating WNT2B
Source: Stem Cell Res Ther. 2019 Feb 22;10:67. doi: 10.1186/s13287-019-1161-9 (PMC6387535; doi:10.1186/s13287-019-1161-9)
Supplement: Supplementary file 1 — Table S1. Sequences of PCR primers used in this study. Figure S1. Flow cytometry analysis of HBMSCs. Figure S2. Quantitative analysis of ALP staining and ARS staining after interference or overexpression of LINC00707 in transfected HBMSCs after 7 days osteogenic induction. Figure S3. (A and B) Quantitative analysis of ARS staining and ALP staining in HBMSCs co-transfected with LINC00707/NC and miR-370-3p mimics/mimics NC after osteogenic induction for 7 days. (C and D) ARS staining and ALP staining in HBMSCs co-transfected with LINC00707/NC and miR-370-3p mimics/mimics NC after osteogenic induction for 7 days. Figure S4. ARS staining and ALP staining in HBMSCs transfected with si-WNT2B after oncogenic induction for 7 days. (DOCX 511 kb) [file 13287_2019_1161_MOESM1_ESM.docx]

**Long noncoding RNA LINC00707 promotes osteogenesis of** **human bone marrow-derived mesenchymal stem cells via the miR-370-3p/WNT2B axis.**

Bo Jia^1^, Zhiping Wang^1^, Xiang Sun^1^, Jun Chen^1^, Jianjiang Zhao^1^, Xiaoling Qiu^2^

1Department of Oral Surgery, Stomatological Hospital, Southern Medical University, 510280 Guangzhou, China.

2 Department of General consulting and Emergency, Stomatological Hospital, Southern Medical University, 510280, Guangzhou, China.

Correspondence to: Professor Xiaoling Qiu, Department of General consulting and Emergency, Stomatological Hospital, Southern Medical University, 366 South Jiang Nan Road, haizhu, guangzhou, guangdong 510280, P.R. China. E-mail: [linglingjojo@163.com](mailto:linglingjojo@163.com)

**
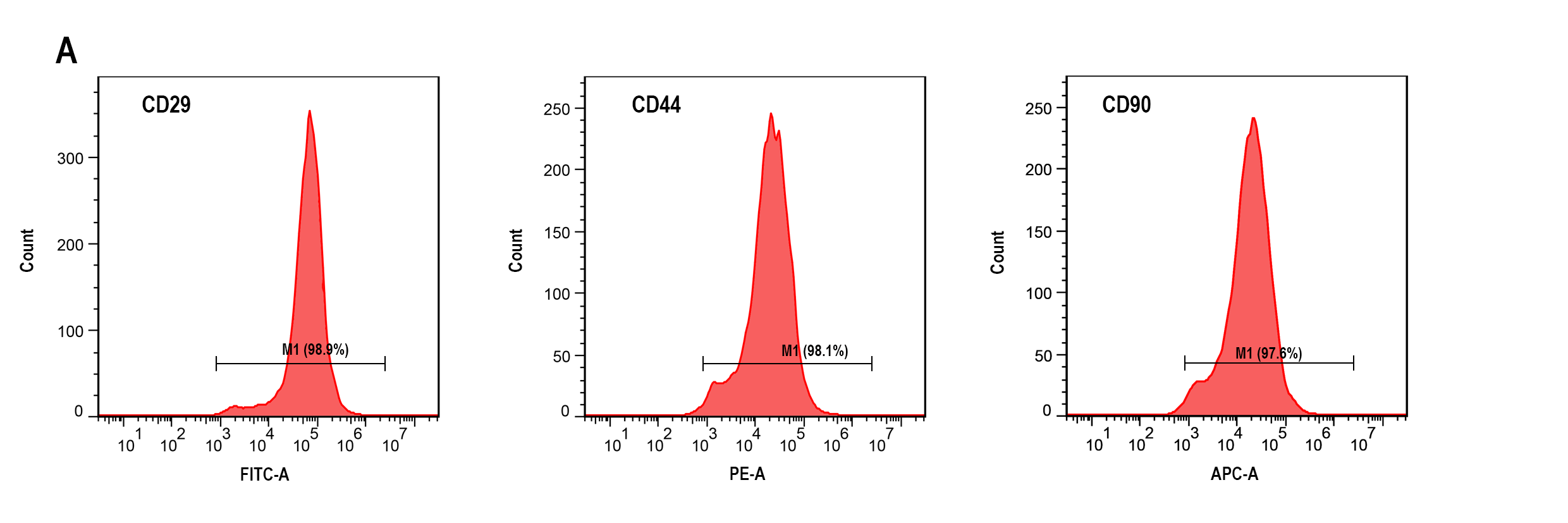
**

**Figure S1** Flow cytometry analysis of HBMSCs. CD marker analysis by flow cytometry revealed that 98.9%, 98.1% and 97.6% of cells were positive for BMSC markers CD29, CD44 and CD90, respectively.

**
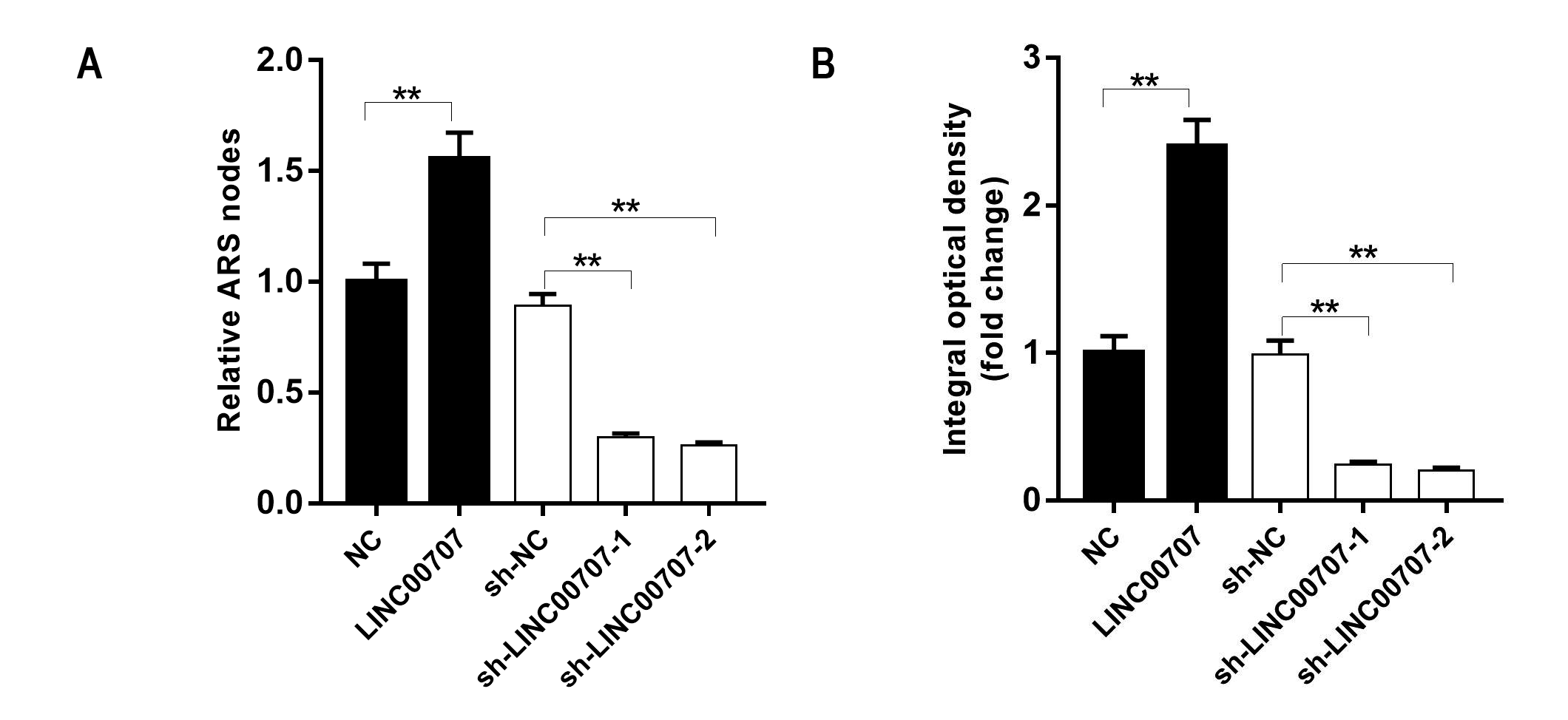
**

**Figure S2** Quantitative analysis of ALP staining and ARS staining after interference or overexpression of LINC00707 in transfected HBMSCs after 7 days osteogenic induction.


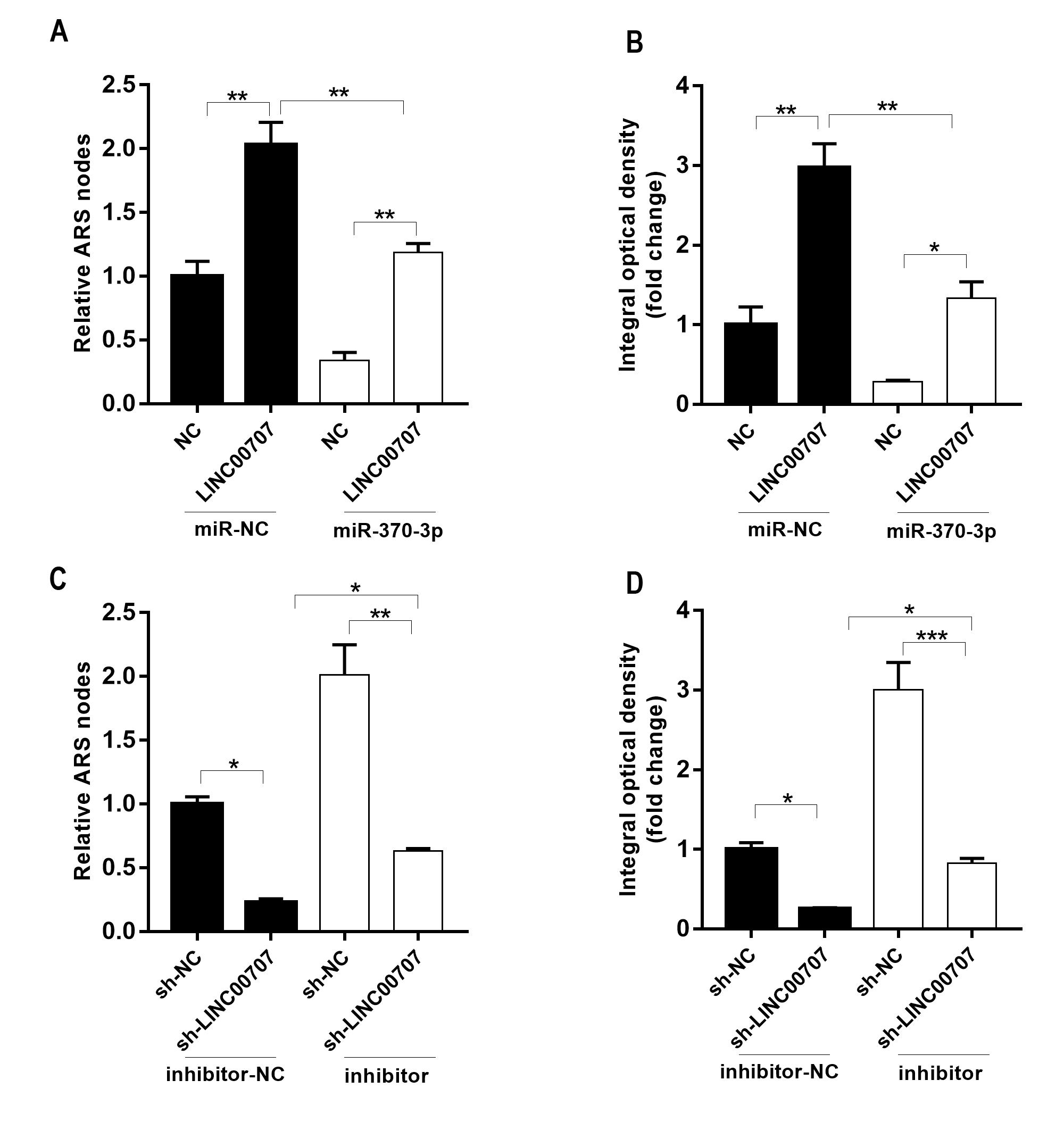


**Figure S3** (A and B) Quantitative analysis of ARS staining and ALP staining in HBMSCs co-transfected with LINC00707/NC and miR-370-3p mimics/mimics NC after osteogenic induction for 7 days. (C and D) ARS staining and ALP staining in HBMSCs co-transfected with LINC00707/NC and miR-370-3p mimics/mimics NC after osteogenic induction for 7 days.


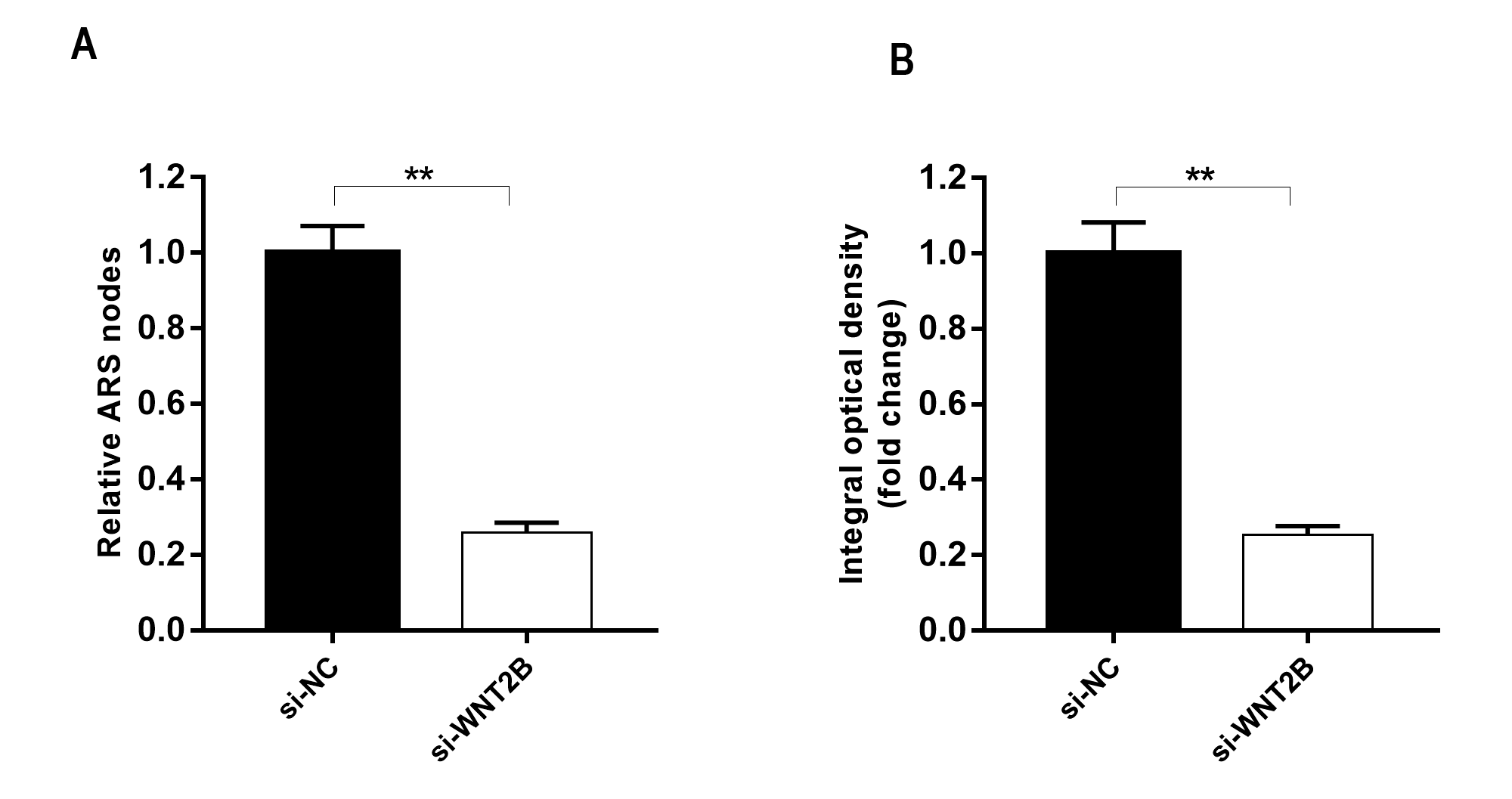


**Figure S4** ARS staining and ALP staining in HBMSCs transfected with si-WNT2B after oncogenic induction for 7 days.

**Table S1** Sequences of PCR primers used in this study.

| LINC00707 | Forward(5’-3’) | TGGAAAGTAAGCCTATTACATATAC |
| --- | --- | --- |
|  | Reverse(5’-3’) | GGTATCACCAACAACCCTGA |
| RP11-348J24.2 | Forward(5’-3’) | CACCGTCCAGCCACAAACC |
|  | Reverse(5’-3’) | AATCCCTACTGCTGTATGGCATC |
| lnc_1369 | Forward(5’-3’) | ACCAGTTACTTTGTGCCATACC |
|  | Reverse(5’-3’) | TTTCAAGAAAGGATCCAATGG |
| lnc_554 | Forward(5’-3’) | TCTTCAGATGCCTCCTGTGGT |
|  | Reverse(5’-3’) | AACCTGGTTAGACCTTGAGTG |
| lnc_1269 | Forward(5’-3’) | ATGAAGGGCACGGCATAGGT |
|  | Reverse(5’-3’) | TGACGCTCGGAAGACAACG |
| lnc_1307 | Forward(5’-3’) | AGATTCCAGTGAGATTGAGGGTC |
|  | Reverse(5’-3’) | TTATCAGAAGGCATAGCACCC |
| β-actin | Forward(5’-3’) | AATCGTGCGTGACATTAAGGAG |
|  | Reverse(5’-3’) | ACGTGTTGGCGTAACAGGTCTT |
| RUNX2 | Forward(5’-3’) | GGCGGGTAACGATGAAAAT |
|  | Reverse(5’-3’) | TCTGTAATCTGACTCTGTCCTTGTG |
| ALP | Forward(5’-3’) | GACGCTGGGAAATCTGTGG |
|  | Reverse(5’-3’) | ATGAAGTGGGAGTGCTTGTATCT |
| OCN | Forward(5’-3’) | CCTCACACTCCTCGCCCTAT |
|  | Reverse(5’-3’) | CTCCTGAAAGCCGATGTGGT |
| WNT2B | Forward(5’-3’) | GCTGGACCAAACCTGAAC |
|  | Reverse(5’-3’) | CAAGAAGTATCGGGAAGC |
| MiR-370-3p | Forward(5’-3’) | CTGCTGGGGTGGAAC |
|  | Reverse(5’-3’) | GGTCCAGTTTTTTTTTTTTTTTACCA |
